# Chronic lymphocytic inflammation with pontine perivascular enhancement responsive to steroids, a mimicker of malignancy: a case report and review of the literature

**DOI:** 10.1186/s13256-021-02814-5

**Published:** 2021-05-18

**Authors:** Eric Zhuang, Lisa Shane, Nima Ramezan, Ameera F. Ismail, Nilesh L. Vora

**Affiliations:** 1 Department of Medicine, University of California Irvine, 101 The City Drive South, Orange, CA 92688 USA; 2 Long Beach Memorial Medical Center, Long Beach, CA USA; 3 Todd Cancer Institute, Long Beach, CA USA

**Keywords:** CLIPPERS, Chronic lymphocytic inflammation with pontine perivascular enhancement responsive to steroids, Lymphohistiocytic inflammation, Lung cancer, Glucocorticoids, Immunosuppression

## Abstract

**Background:**

Chronic lymphocytic inflammation with pontine perivascular enhancement responsive to steroids is a clinically and radiographically distinct inflammatory syndrome affecting multiple structures of the brain, including the cerebellum, brainstem, and spinal cord. The clinical presentation can be variable, including ataxia, nystagmus, dysarthria, dysphagia, and other subacute brainstem, cranial nerve, or cerebellar symptoms. These symptoms can be subacute to chronic, episodic, and progressive, making the diagnosis challenging. The hallmark radiographic magnetic resonance imaging findings are gadolinium-enhancing punctate lesions predominantly “peppering” the pons in a perivascular pattern.

**Case presentation:**

Here, we describe a case and literature review of a 74-year-old Caucasian male who presented with subacute symptoms of ataxia, diplopia, and generalized fatigue. Physical examination was notable for horizontal nystagmus and wide-based gait. Magnetic resonance imaging revealed angiocentric enhancement predominantly in the brainstem and cerebellum, with involvement of the basal ganglia, thalami, and supratentorial white matter. Meanwhile, a screening computed tomography scan demonstrated a right upper lobe mass with biopsy proving primary lung cancer. Biopsy of one of the brain lesions showed perivascular infiltrate primarily composed of CD3+ T cells, scattered CD20+ B cells, and no signs of malignancy. The patient was started on high-dose glucocorticoids followed by a maintenance regimen with rapid improvement clinically and radiographically. Given extensive work-up was negative, these clinical and radiographic findings were consistent with chronic lymphocytic inflammation with pontine perivascular enhancement responsive to steroids.

**Conclusions:**

This case illustrates the difficulty of diagnosing chronic lymphocytic inflammation with pontine perivascular enhancement responsive to steroids, given its variable presentation, lack of specific laboratory findings, and poorly understood pathogenesis. We demonstrate a case that responded well to oral corticosteroid burst followed by a taper to the lowest corticosteroid dose clinically possible. Failure to recognize this syndrome could result in permanent central nervous system morbidity. Therefore, earlier recognition is crucial for this treatable condition.

## Background

Chronic lymphocytic inflammation with pontine perivascular enhancement responsive to steroids (CLIPPERS) is a clinically and radiologically distinct pontine-predominant encephalomyelitis with marked glucocorticoid responsiveness. This was first described in 2010 by Pittock *et al.* after eight patients presented with similar clinical and magnetic resonance imaging (MRI) findings: subacute diplopia, ataxia, facial paresthesia, and punctate and curvilinear gadolinium-enhancing lesions “peppering the pons.” Nystagmus, dysarthria, dysphagia, and other subacute brainstem, cranial nerve, or cerebellar symptoms occurred. Biopsy of lesions typically demonstrate perivascular lymphohistiocytic infiltrate without granulomas, infection, lymphoma, or vasculitis [1]. Clinical and radiological response follows the administration of glucocorticoid or other immunosuppressive therapy [2, 3]. Characteristics of CLIPPERS are summarized in Table 1. The overall course of the disease appears to be favorable; however, most patients routinely worsen following glucocorticoid taper and require chronic glucocorticoid therapy or other immunosuppressive therapy [4]. In patients not on chronic steroid therapy or other immunosuppressive agents, the disease has a relapsing–remitting course, with an approximate mean annual relapse rate (ARR) of 0.5 (range, 0.25–2.8) [2]. Clinical and radiological improvement is nearly systematically observed following readministration of high doses of steroids. Since often no progressive morbidity occurs following immunosuppressive treatment, the clinical and radiological sequelae that persist may be due to previously unrecognized or untreated relapses. This suggests that maintaining this disease in remission may prevent the accumulation of disability. Multiple reports observed no further relapses when chronic corticosteroid therapy was maintained above 20 mg/day [4, 5]. We present a case of CLIPPERS in which the diagnosis was complicated, given the patient’s lung cancer, and we underline the successful diagnosis, treatment, and disease course.

**Table 1:** Core features and proposed diagnostic criteria for chronic lymphocytic inflammation with pontine perivascular enhancement responsive to steroids (adapted from Tobin *et al.* [7])

Clinical
Subacute pontocerebellar dysfunction with or without other CNS symptoms such as cognitive dysfunction and myelopathy
CNS symptoms responsive to corticosteroid therapy
Absence of peripheral nervous system disease
Lack of alternative explanation for clinical presentation
Radiological
MRI with homogeneous, gadolinium-enhancing nodules without ring enhancement or mass effect predominating in the pons or cerebellum measuring < 3 mm
Marked improvement in gadolinium enhancement after corticosteroid treatment
Homogeneous T2 signal abnormality where degree of T2 does not significantly exceed the size or area of post gadolinium enhancement
Spinal lesions with similar T2 and gadolinium-enhancing lesions as above
Pathological
Lymphocytic inflammation with perivascular predominance and parenchymal diffuse infiltration
T cells predominating infiltration with variable macrophage components
Absence of myelin loss or focal secondary myelin loss
Lack of alternative explanation for pathological presentation
Definite CLIPPERS: patient fulfilling all clinical, radiological, and pathological criteria
Probable CLIPPERS: patient fulfilling all clinical and radiological criteria without available pathology
Differential diagnoses, including CNS lymphoma, lymphomatoid granulomatosis, glioma, primary CNS vasculitis, paraneoplastic syndrome, neurosarcoidosis, demyelinating disease, acute disseminated encephalomyelitis (ADEM), neuromyelitis optica (NMO) neuro-Behcet’s disease, Sjögren’s syndrome, tuberculosis, Whipple’s disease, and histiocytosis, should be excluded

## Case presentation

A 74-year-old Caucasian male presented to his primary care physician with a 3-month history of ataxia, diplopia, and generalized fatigue. He had a medical history of hypertension, coronary artery disease, and peripheral vascular disease. On examination, the patient had a wide-based gait with postural instability, and horizontal nystagmus. He had normal reflexes, no focal motor deficits, no focal muscle atrophy, no focal sensory deficits, and no localized deafness or tinnitus. MRI of the brain with contrast was performed, which revealed numerous 1–2 mm intraaxial fluid-attenuated inversion recovery (FLAIR) hyperintense lesions scattered predominantly throughout the central brain, with several periventricular and subcortical lesions concerning for possible central nervous system (CNS) lymphoma, metastatic cancer, or an atypical granulomatous, inflammatory, or infectious etiology. The patient was subsequently admitted for further work-up.

Lumbar puncture showed mildly elevated protein at 64 mg/dL but was otherwise unremarkable. ´There were no oligoclonal bands or malignant cells. Laboratory work including antinuclear antibody (ANA), anti-double stranded deoxyribonucleic acid (DNA) (anti-dsDNA) antibody, anti-single-stranded DNA (anti-ssDNA) antibody, antimitochondrial antibody, and serum angiotensin-converting enzyme (ACE) were unremarkable. The patient also tested negative for *Coccidioides*, toxoplasmosis, *Cryptococcus*, Q fever, *Rickettsia typhi*, *Borrelia burgdorferi*, syphilis, *Mycobacterium tuberculosis*, cytomegalovirus (CMV), Epstein–Barr virus (EBV), and human immunodeficiency virus (HIV). The patient underwent computed tomography (CT) imaging for malignancy work-up, which revealed a spiculated right upper lobe lung mass compatible with lung cancer.

**Fig. 1:**
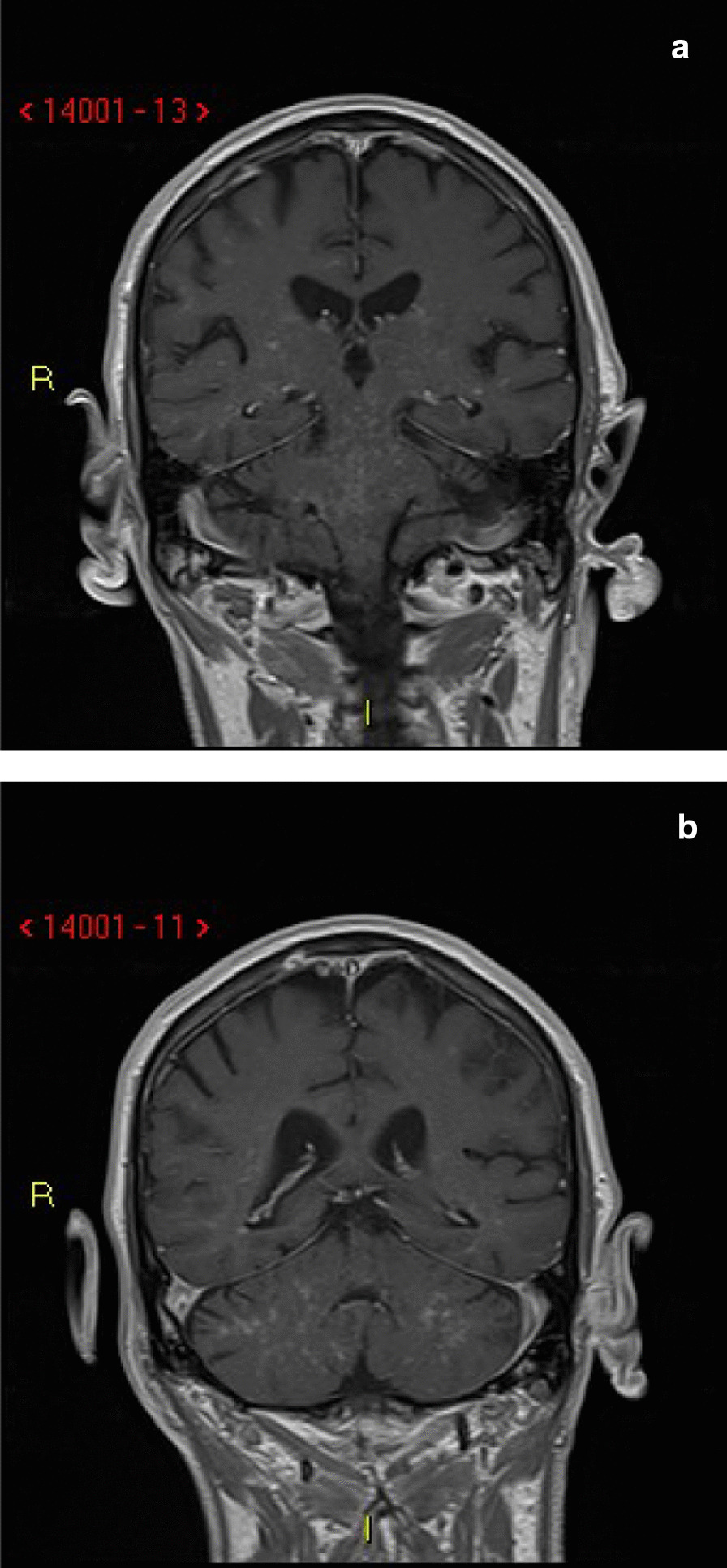
**a**, **b** Coronal magnetic resonance imaging T1 postcontrast images demonstrating angiocentric enhancement predominantly in the brainstem and cerebellum with involvement of the basal ganglia, thalami, and supratentorial white matter

CT-guided biopsy of the lung mass revealed primary lung squamous carcinoma. The patient underwent staging positron emission tomography (PET)/CT that showed intense fluorine-18 deoxyglucose (FDG) accumulation at the site of the patient’s known lung cancer but no pathological thoracic or mediastinal lymph nodes or any other sites suggestive of distant metastatic disease. There was relatively symmetrical FDG accumulation into the cerebral cortices, subcortical gray matter, and cerebellar hemispheres. An MRI brain stealth protocol with contrast was performed and demonstrated angiocentric enhancement predominantly in the brainstem and cerebellum, with involvement of the basal ganglia, thalami, and supratentorial white matter (Fig. 1). This raised suspicion for CLIPPERS; however, the differential diagnosis also included neurosarcoidosis, lymphomatoid granulomatosis, angiocentric lymphoma, vasculitis, cerebral amyloid disease, and leptomeningeal carcinomatosis, though less likely given the lack of leptomeningeal enhancement.

**Fig. 2:**
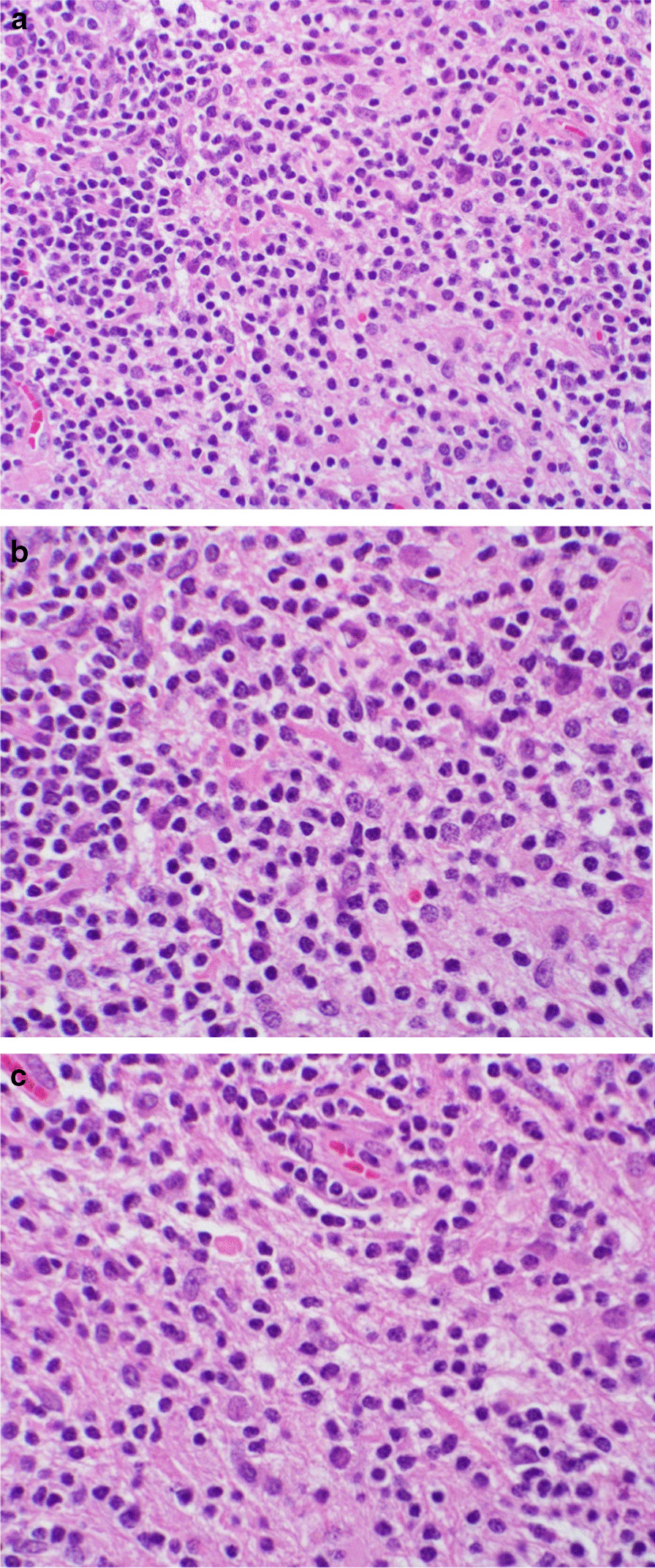
Sections of cerebellum. **a** Lymphohistiocytic infiltrate. **b** Higher power shows small “mature” lymphocytes and admixed histiocytes. No well-formed granuloma or immature cell population. **c** Inflammatory cells surrounding a small vessel

**Fig. 3:**
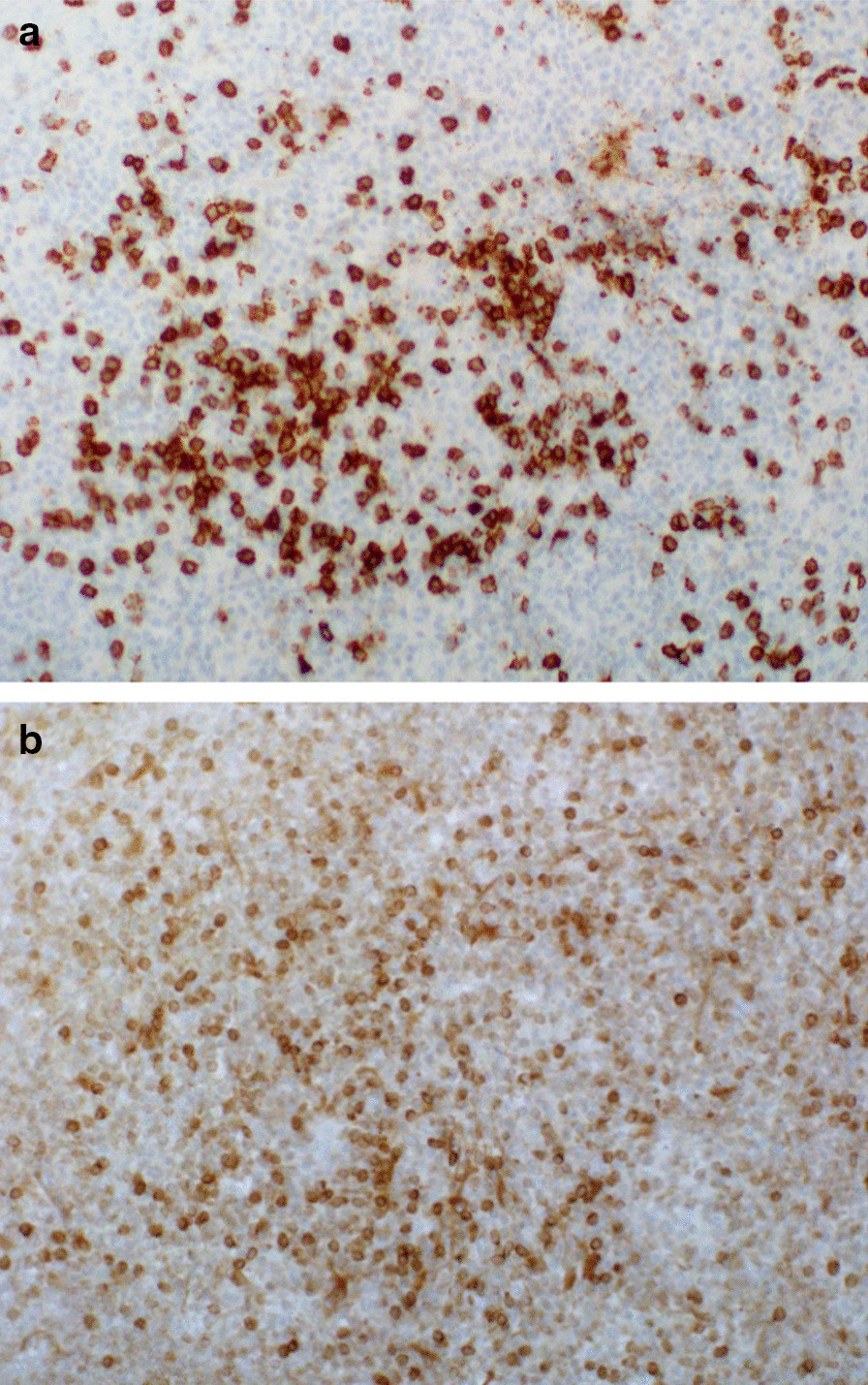
**a** CD20 highlighting lesser numbers of B lymphocytes. **b** CD3 highlighting predominance of T lymphocytes

Neurosurgery was consulted, and a brain biopsy was planned. A cerebellar biopsy was performed, given it was the area with the highest density of enhancement. The specimen showed perivascular infiltrates of lymphocytes (Fig. 2). No malignant cells or neutrophils were seen. Immunohistochemical staining revealed mostly CD3+ T cells with scattered CD20+ B cells (Fig. 3). Given the clinical, radiological, and pathological findings were consistent with CLIPPERS,the patient was started on an empiric trial of steroid therapy: prednisone 40 mg oral daily.

On follow-up a month later, the patient reported that he had felt “back to himself.” He reported the ataxia, diplopia, and generalized fatigue were significantly improved, though not yet completely resolved. This was confirmed on physical examination. After 2 months on steroid therapy, MRI of the brain showed dramatic improvement radiologically, with the enhancing lesions throughout the brain seen on prior studies resolved. Clinically the patient reported he was “doing amazingly well,” his ataxia had resolved, and he was no longer requiring a cane or walker to ambulate. Therefore, the patient had done well on steroids. It was determined that the patient should be tapered off steroids; the patient tapered off steroids over 1 month, for a total of 3 months on steroid therapy.

As the brain biopsy was negative for malignant cells, and the PET/CT demonstrated no overt signs of any other metastatic disease, the patient’s lung cancer was clinically stage IA. He subsequently underwent robotic right upper lobectomy with hilar and mediastinal lymph node dissection. Surgical findings demonstrated T1bN0 disease, consistent with pathological stage IA2 lung cancer. No adjuvant therapy was indicated, and surveillance CT scans as of 1 year have demonstrated no recurrence.

Four months after stopping steroid therapy, the patient began to have a return of ataxia, and during this episode he also developed intermittent expressive aphasia that would spontaneously resolve after several hours. MRI of the brain with contrast was performed and demonstrated reappearance of the multiple lesions in the brain. This was concerning for relapsed CLIPPERS. The patient was treated with intravenous dexamethasone 4 mg every 6 hours over 1 day, followed by a maintenance regimen of prednisone 20 mg once daily. On 2-month follow-up, the patient had no further events or symptoms and MRI of the brain showed improvement of the lesions in the brain.

## Discussion and conclusion

We encountered a patient whose clinical and radiological findings were concerning for metastatic lung cancer; however, the patient was found to have CLIPPERS. This case highlights two important issues. The first is the challenging nature of diagnosing CLIPPERS. The second is the treatment regimen.

To date, validated diagnostic criteria for CLIPPERS are not available, and thorough work-up is mandatory to exclude alternative conditions (Table 1) including vascular, inflammatory, infectious, neoplastic, and paraneoplastic conditions that can overlap with CLIPPERS syndrome. Several papers have proposed criteria to make the diagnosis [2, 4, 6, 7]. Simon *et al.* highlighted clinical, radiological, corticosteroid response, and histopathological features as diagnostic criteria. Taieb *et al.* have characterized CLIPPERS as subacute episodic brainstem signs and symptoms, punctate and curvilinear enhancing lesions mainly involving the pons, prompt clinical and radiological steroid sensitivity, and in the absence of alternative diagnosis. Tobin *et al.* have published criteria modified from Simon *et al.*, where they propose “definite CLIPPERS,” defined as a patient who fulfills all clinical, radiological and neuropathological criteria, and “probable CLIPPERS,” defined as a patient who fulfills all clinical and radiological criteria without available neuropathology.

This patient presented with subacute symptoms of ataxia, diplopia, and generalized fatigue. The neurological examination was important in differentiating central and peripheral nervous system disease in this patient. He demonstrated a wide-based gait with postural instability and horizontal nystagmus with a lack of deafness or tinnitus, suggestive of a central etiology [8]. Given the patient’s lung cancer was discovered around the same time as the brain lesions, it was important to exclude metastatic malignancy, which would require a different therapeutic approach. With no specific biomarker for CLIPPERS, we felt brain biopsy was necessary prior to initial treatment to exclude another disease entity that would require alternative treatment. Given our patient demonstrated clinical signs of ataxia and diplopia, radiological signs of punctate enhancing lesions involving the pons, pathological perivascular lymphocytic inflammation, absent signs of peripheral nervous system disease, and prompt clinical and radiological steroid responsiveness, this case is likely a case of “definite CLIPPERS” as defined by Tobin *et al.*

It is possible that CLIPPERS could be a marker, a paraneoplastic marker of malignancy. CLIPPERS has been reported in cases of lymphoma, before the emergence of lymphoma, preceding colon cancer, and after lymphoma remission [9–16]. Taieb *et al.* suggests that, in the setting of lymphoma, the presence of CLIPPERS features could relate to (1) paraneoplastic vasculitis involving small vessels, (2) lymphoma cell infiltration, and (3) sentinel lesions. Sentinel lesions, considered as host immunity fighting against cancer, have been described as responsive to steroids preceding primary CNS lymphoma. However, primary CNS lymphoma, unlike CLIPPERS, is often associated with rapid disease progression and death, after an initial but transient improvement with steroids [17]. Whether CLIPPERS is truly an independent disease entity or a manifestation of another systemic disease remains uncertain.

In the absence of randomized placebo-controlled trials, formal recommendations of the optimal treatment modality for CLIPPERS are yet to be determined. This is likely due to the relatively few patients with CLIPPERS reported so far. A number of papers have proposed the initial treatment should begin with high-dose steroids, such as a course of intravenous high-dose methylprednisolone daily for 5 days [1, 2, 4, 5, 18–20]. This is followed by an oral maintenance corticosteroid regimen that is slowly tapered to an equivalent of prednisone 20 mg/day. Of important note, tapering the steroid regimen to below 10–20 mg/day frequently leads to inevitable relapse [5]. The patient illustrated in this case underwent initial treatment with prednisone 40 mg followed by a slow taper to cessation over 3 months with significant clinical and radiological improvement. Time from symptom onset to corticosteroid initiation was approximately 76 days. As predicted, after withdrawal of steroid therapy, the patient relapsed. During this relapse, the patient was administered dexamethasone 16 mg intravenously over 1 day with rapid improvement, followed by a maintenance regimen of prednisone 20 mg/day. The successful treatment of the disease during the acute phase with prednisone 40 mg oral daily and later exacerbation with dexamethasone 4 mg every 6 hours over 1 day, followed by a prednisone oral taper, suggests that high-dose steroids over 5 days may not be necessary and a shorter initial course of high-dose steroids may be adequate in some cases. After a short course of high-dose steroid therapy, the patient remained stable on maintenance steroid therapy of prednisone 20 mg once per day, and so chronic long-term immunosuppressive therapy appears to be warranted to keep CLIPPERS in remission. Based on other clinical observations, steroid-sparing treatment has also been proposed using methotrexate, cyclophosphamide, hydroxychloroquine, or azathioprine [2].

Overall, CLIPPERS represents a distinct clinical, radiological, and pathological process that can be diagnosed after exclusion of other differential diagnoses. However, the pathogenesis of CLIPPERS is still unknown, and it remains unclear whether it represents a separate and independent disease entity or a syndrome with heterogeneous etiologies. For now, the primary treatment is with high-dose corticosteroids. Physicians must be aware of this condition and differential diagnoses, given it has been shown that early diagnosis and corticosteroid-based treatment can prevent irreversible morbidity. Our observations are consistent with previous reports that treatment with corticosteroids leads to prompt clinical and radiological improvement, and the cessation of therapy leads to relapse. Further studies to determine the pathophysiology, potential biomarkers, and reliable diagnostic criteria, as well as the optimal form and duration of treatment, are necessary.

## Data Availability

Not applicable.

## Abbreviation

CLIPPERS: Chronic lymphocytic inflammation with pontine perivascular enhancement responsive to steroids
